# Delirious Mania in an elderly person?: a case report.

**DOI:** 10.1192/j.eurpsy.2023.1992

**Published:** 2023-07-19

**Authors:** P. Setién Preciados, E. Arroyo Sánchez

**Affiliations:** 1 Psychiatry, Hospital Universitario Príncipe de Asturias, Alcalá de Henares, Spain

## Abstract

**Introduction:**

Delirious mania is a potentially fatal neuropsychiatric syndrome of unknown etiology often characterized by the acute onset of delirium, symptoms of mania, and psychosis. The presentation is often punctuated by catatonia.

Delirious mania may constitute up to 15% of all acute mania cases. When delirious mania is unrecognized or improperly treated, it can progress rapidly in severity and can become life-threatening.

Despite being relatively prevalent, literature on delirious mania is sparse, and there are no formal diagnostic criteria or treatment guidelines.

**Objectives:**

Review delirious mania as an entity, its symptoms, type of patient and treatment.

**Methods:**

Presentation of a patient’s case and review of existing literature regarding delirious mania and its characteristics.

**Results:**

In delirious mania symptoms present abruptly, within hours. Symptomatology varies from psychotic (hallucinations, delusions…), maniac (agitation, dysphoria…) and altered sensorium (desorientation, fluctuation of symptoms…). A differential diagnosis has to be done, as well as discarding an organic origin, which in the end, as illustrated in this case, was the etiology of the symptomatology in this patient.

**Conclusions:**

Delirious mania is a clinical entity very underdiagnosed given that patients exhibit an array of different symptoms, making diagnosis very challenging for professionals. It should always be considered in differential diagnosis when these symptoms are present, especially in elderly people, given that early treatment is key. However, discarding an orgnanic origin should always be the first thing to do in clinical practice.

**Disclosure of Interest:**

None Declared

